# Supplementary material for: The Association between New World Alphasatellites and Bipartite Begomoviruses: Effects on Infection and Vector Transmission
Source: Pathogens. 2021 Sep 26;10(10):1244. doi: 10.3390/pathogens10101244 (PMC8538204; doi:10.3390/pathogens10101244)
Supplement: Supplementary file 1 [file pathogens-10-01244-s001.zip › pathogens-1297685-supplementary.pdf]

Supplementary Table S1. Primers used for conventional and quantitative real-time PCR.

Primer name	Sequence (5'-3')	C/Q*	Tm	Detection
ToSRV-A(For)	CAGTAGTTGCCCTCAAATTGAAG	C	66°C	ToSRV DNA-A
ToSRV-A(Rev)	CACGTGTAGCAATCTCCTTAAAGAG	C	66°C	ToSRV DNA-A
ToYSV-A(For)	CACTTCGTTCGAGAAAACCTTTGAG	C	66°C	ToYSV DNA-A
ToYSV-A(Rev)	CATGAGGGGACCATCAAGG	C	66°C	ToYSV DNA-A
1978(For)	GCATCTGCAGGCCCACATYGTCTTYCCNGT	C	55°C	EuYMV DNA-A
496(Rev)	GGCTTYCTRTACATRGG	C	55°C	EuYMV DNA-A
EuYMA(For)	GATCGGATCCATATGGGCTCAGCC	C	65°C	EuYMA alpha-Rep
EuYMA(Rev)	GATCGAGCTCTTATTCCATATACGC	C	65°C	EuYMA alpha-Rep
ToYSA (rep-sat)(For)	GCAAGGGAGCAACAAGAGGA	C	66°C	ToYSA alpha-Rep
ToYSA (rep-sat)(Rev)	AAGAAGGCAAGATGGTAGCCC	C	66°C	ToYSA alpha-Rep
qToSRV-A(For)#	AAAGTAAAGTGATTGTCTGTGG	Q	60°C	ToSRV DNA-A
qToSRV-A(Rev)#	GCCGTTCAACAAATTGGG	Q	60°C	ToSRV DNA-A
qToYSV-A(For)	CCACGATTTTAAAGCTGCATTCT	Q	60°C	ToYSV DNA-A
qToYSV-A(Rev)	CAATCCTGGTGAGGGAGTCAGT	Q	60°C	ToYSV DNA-A
qEuYMV-A(For)&	AAGGCCTCTTCATGGGTGAA	Q	60°C	EuYMV DNA-A
qEuYMV-A(Rev) &	TTCGGTACATCTGGGCCTCTA	Q	60°C	EuYMV DNA-A
qEuYMA (For) &	CAAGAGGAAGACGATGGAACGT	Q	60°C	EuYMA alpha-Rep
qEuYMA(Rev) &	AGCAGCGACGATACAGCTTAGG	Q	60°C	EuYMA alpha-Rep
qToYSA(For)	TGGGTGTATGGCTCTCAA	Q	64°C	ToYSA alpha-Rep
qToySA(Rev)	CTTGATGTTCTCTCCCTTTCC	Q	64°C	ToYSA alpha-Rep

* C, conventional PCR; Q, quantitative, real-time PCR. # From Silva et al., 2014. & From Mar et al., 2017.

Supplementary Table S2. Infectivity of three New World bipartite begomoviruses (euphorbia yellow mosaic virus, EuYMV; tomato severe rugose virus, ToSRV; tomato yellow spot virus, ToYSV), alone or in the presence of euphorbia yellow mosaic alphasatellite (EuYMA), in tomato (*Solanum lycopersicum*) and *Nicotiana benthamiana*.

	**Tomato**						***Nicotiana benthamiana***					
	**Exp. I**		Exp. II		**Total**		Exp. I		Exp. II		**Total**	
	**alone**	**+EuYMA**	alone	+EuYMA	alone	+EuYMA	alone	+EuYMA	alone	+EuYMA	alone	+EuYMA
						**EuYMV**						
EuYMV	7/15*	0/15	4/10	6/10	**11/25**	**6/25**	11/11	10/11	4/10	8/8	**15/21**	**18/19**
EuYMA		0/15		6/10		**6/25**		5/11		7/8		**12/19**
						**ToSRV**						
ToSRV	15/15	15/15	6/10	10/10	**21/25**	**25/25**	11/11	6/11	7/7	9/10	**18/18**	**15/21**
EuYMA		8/15		10/10		**18/25**		6/11		8/10		**14/21**
						**ToYSV**						
ToYSV	15/15	15/15	7/10	10/10	**22/25**	**25/25**	11/11	11/11	8/9	9/9	**19/20**	**20/20**
EuYMA		3/15		10/10		**13/25**		10/11		9/9		**19/20**

* Number of PCR-positive plants/number of inoculated plants confirmed at 28 days post-inoculation in two independent experiments (Exp. I and Exp. II).

Supplementary Table S3. Infectivity of three New World bipartite begomoviruses (euphorbia yellow mosaic virus, EuYMV; tomato severe rugose virus, ToSRV; tomato yellow spot virus, ToYSV), alone (-) or in the presence of tomato yellow spot alphasatellite (ToYSA) (+), in tomato (*Solanum lycopersicum*), *Nicotiana benthamiana* and *Leonurus sibiricus*.

	**Tomato**								***Nicotiana benthamiana***						***Leonurus sibiricus***					
	**Exp. I**		**Exp. II**		**Exp. III**		**Total**		Exp. I		Exp. II		**Total**		Exp. I		Exp. II		**Total**	
**ToYSA**	**−**	**+**	**−**	**+**	**−**	**+**	**−**	**+**	**−**	**+**	**−**	**+**	**−**	**+**	**−**	**+**	**−**	**+**	**−**	**+**
				**EuYMV**																
EuYMV	4/10	8/10	5/15	8/15	13/15	11/14	**22/40**	**27/39**	4/10	5/10	9/14	8/13	**13/24**	**13/23**	0/10	0/14	0/13	0/15	**0/23**	**0/29**

ToYSA		8/10		7/15		11/14		**26/39**		2/10		8/13		**10/23**		n.d.[#]		n.d.		**n.d.**
					**ToSRV**															
ToSRV	6/10	7/9	10/14	3/11	7/15	4/16	**23/39**	**14/36**	6/6	5/7	11/15	9/13	**17/21**	**14/20**	0/10	0/10	0/14	0/15	**0/24**	**0/25**
ToYSA		6/9		0/11		1/16		**7/36**		5/7		7/13		**12/20**		n.d.		n.d.		**n.d.**
					**ToYSV**															
ToYSV	7/10	10/10	7/15	5/14	10/15	10/15	**24/40**	**25/39**	7/8	10/10	14/15	15/15	**21/23**	**25/25**	8/14	8/15	11/14	13/15	**19/28**	**21/30**
ToYSA		4/10		1/14		10/15		**15/39**		10/10		12/15		**22/25**		6/15		11/15		**17/30**

* Number of PCR-positive plants/number of inoculated plants confirmed 28 days post-inoculation in three independent experiments in tomato and in two independent experiments in *N. benthamiana* and *L. sibiricus*. [#] n.d., not done.

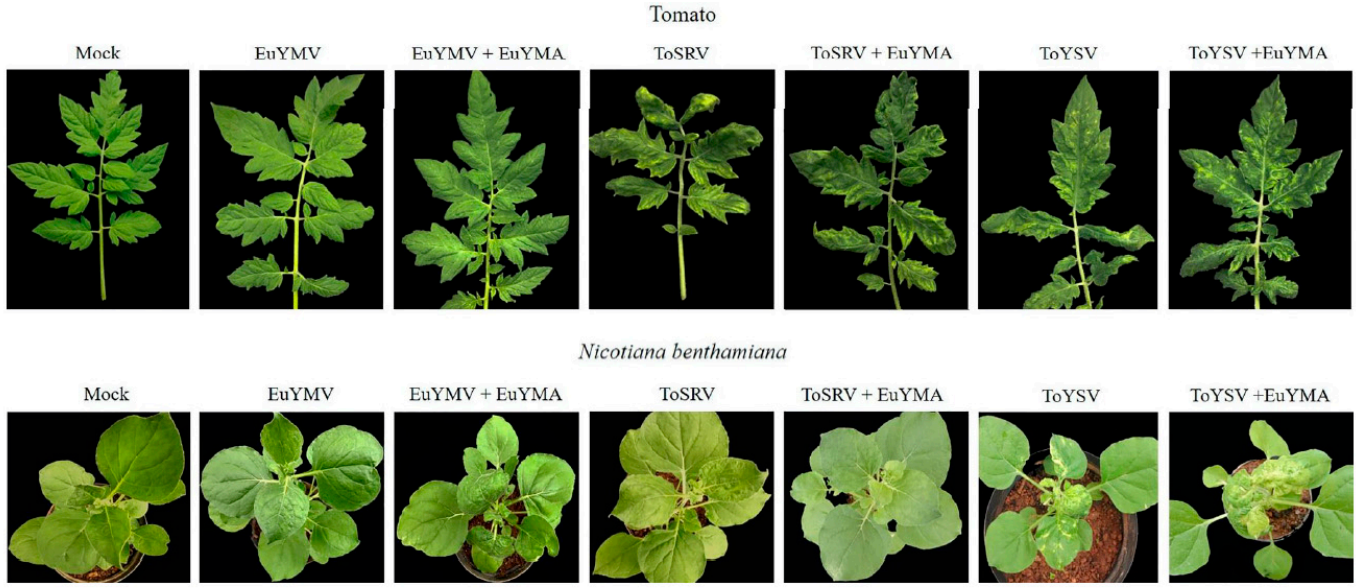

Supplementary Figure S1. Symptoms in tomato and *N. benthamiana* plants infected with the begomoviruses euphorbia yellow mosaic virus (EuYMV), tomato severe rugose virus (ToSRV) and tomato yellow spot virus (ToYSV), in the absence or presence of euphorbia yellow mosaic alphasatellite (EuYMA) at 14 days post-inoculation by biolistics.

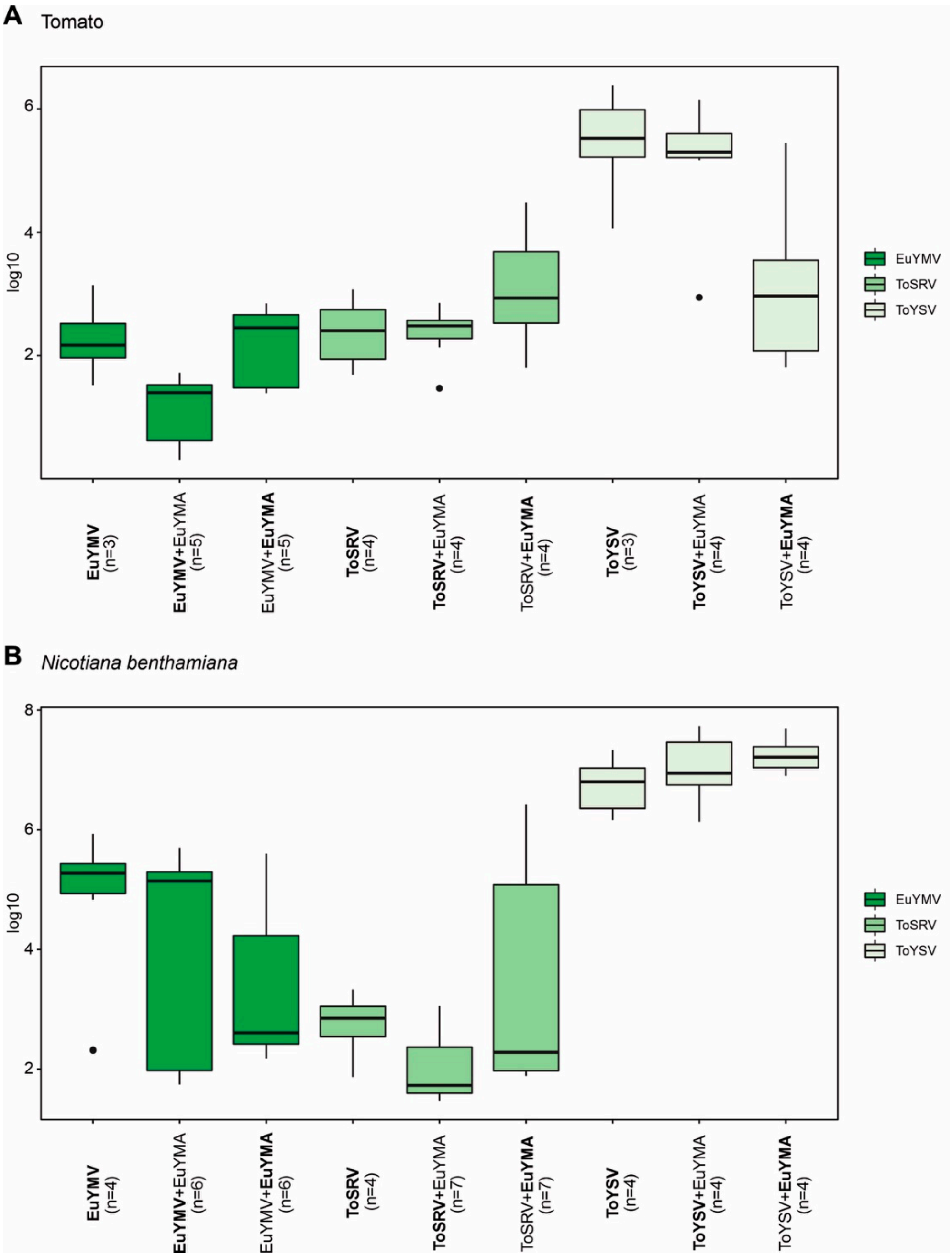

Supplementary Figure S2. Accumulation of euphorbia yellow mosaic virus (EuYMV), tomato severe rugose virus (ToSRV) and tomato yellow spot virus (ToYSV) DNA-A in the absence or presence of euphorbia yellow mosaic alphasatellite (EuYMA). Absolute quantification of viral DNA was performed at 14 days post-inoculation in (**A**) tomato (*S. lycopersicum*) and (**B**) *N. benthamiana*. Boxplots correspond to viral accumulation presented as the log of the number of molecules. The number of plants analyzed in each treatment (*n*) is indicated, and the agent (begomovirus or satellite) evaluated is underlinedin bold.

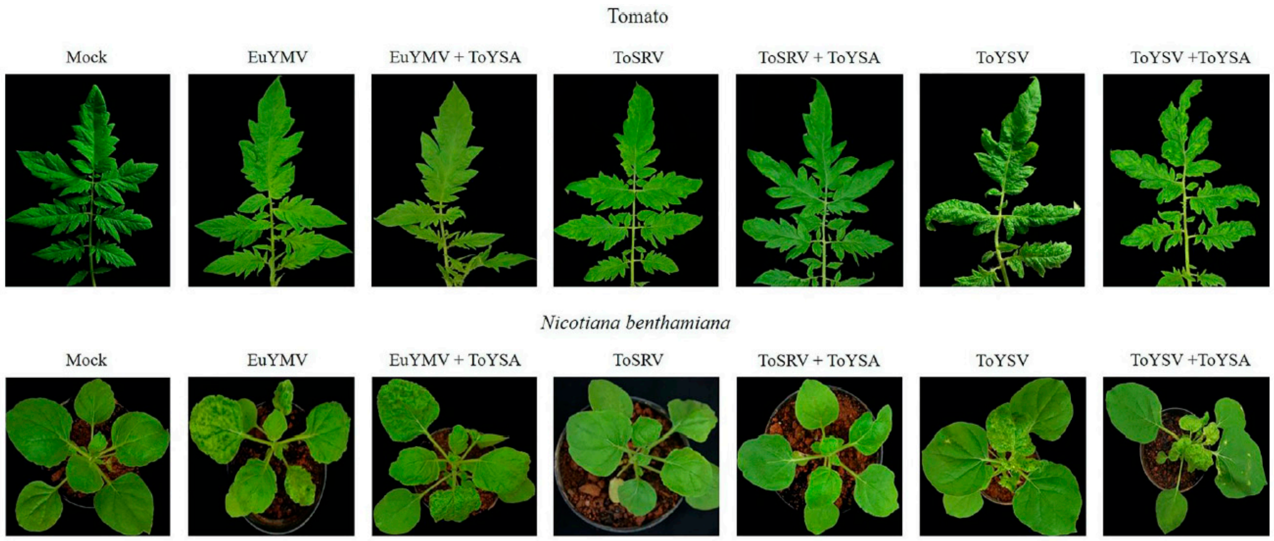

Supplementary Figure S3. Symptoms in tomato and *N. benthamiana* plants infected with the begomoviruses euphorbia yellow mosaic virus (EuYMV), tomato severe rugose virus (ToSRV) and tomato yellow spot virus (ToYSV), in the absence or presence of tomato yellow spot alphasatellite (ToYSA) at 14 days post-inoculation by biolistics.

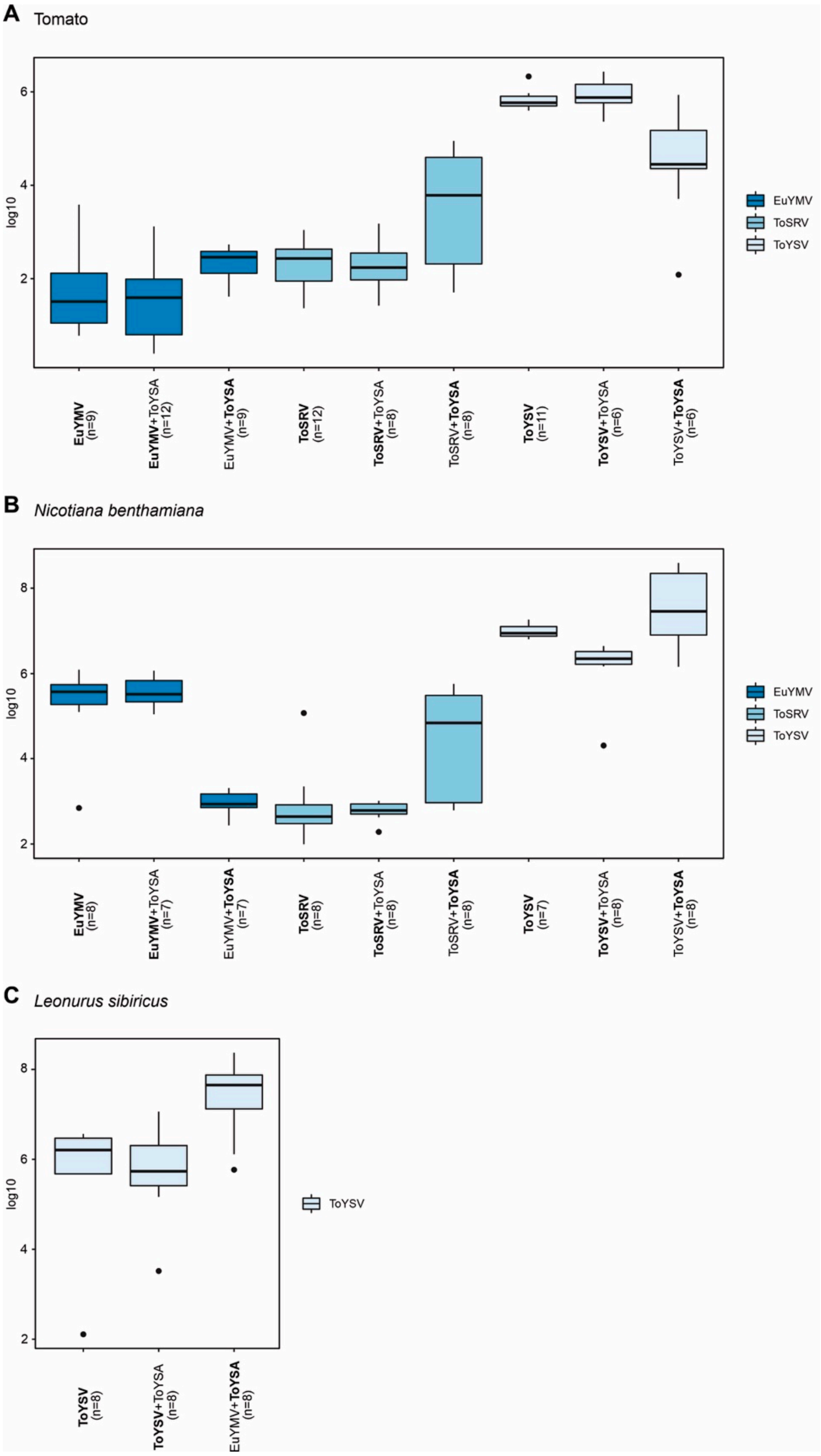

Supplementary Figure S4. Accumulation of euphorbia yellow mosaic virus (EuYMV), tomato severe rugose virus (ToSRV) and tomato yellow spot virus (ToYSV) DNA-A in the absence or presence of tomato yellow spot alphasatellite (ToYSA). Absolute quantification of viral DNA was performed at 14 days post-inoculation in (**A**) tomato (*S. lycopersicum*), (**B**) *N. benthamiana* and (**C**) *L. sibiricus*. Boxplots correspond to viral accumulation presented as the log of the number of molecules. The number of plants analyzed in each treament (*n*) is indicated, and the agent (begomovirus or satellite) evaluated is underlinedin bold.

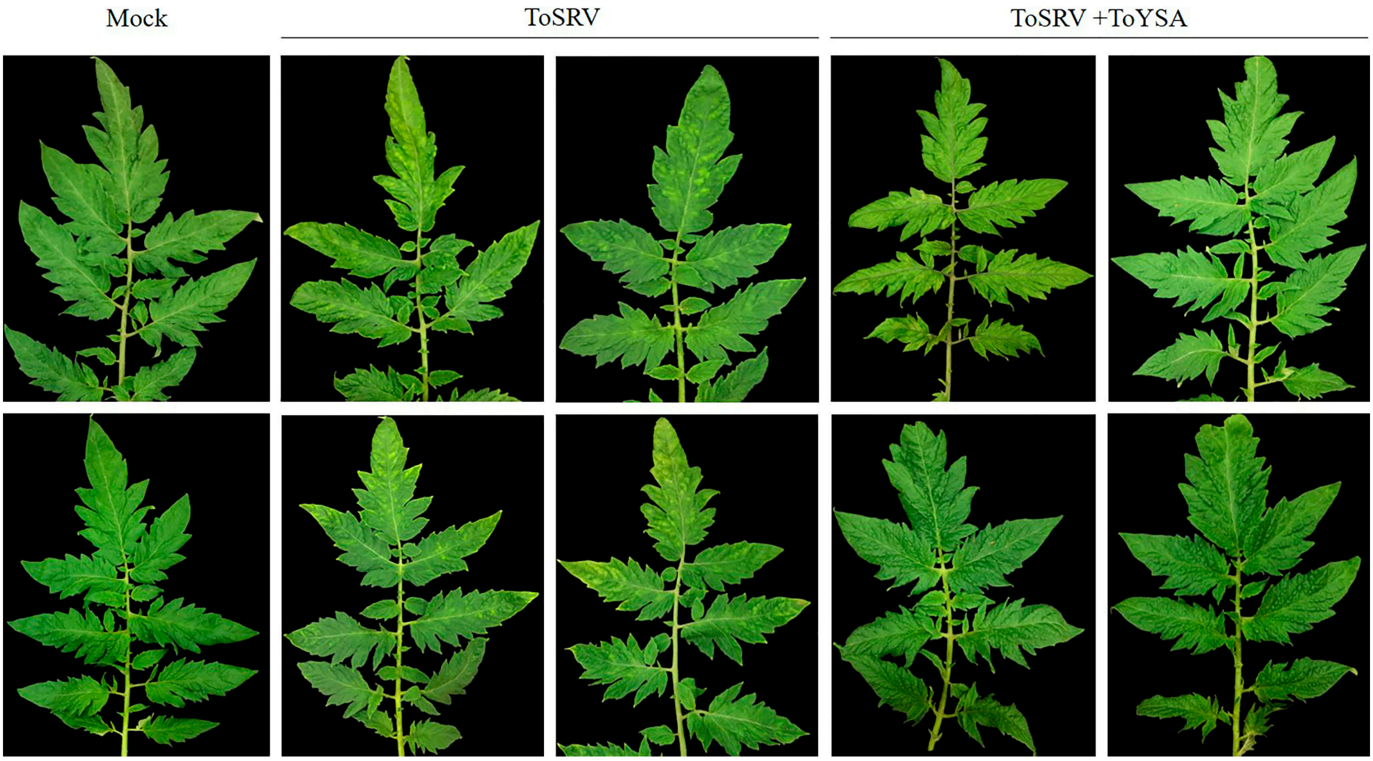

Supplementary Figure S5. Symptoms in tomato plants following whitefly (*Bemisia tabaci* MEAM1) transmission of tomato yellow spot virus (ToYSV), alone or in the presence of tomato yellow spot alphasatellite (ToYSA), at 28 days post-transmission.
